# Proteolytic systems and AMP-activated protein kinase are critical targets of acute myeloid leukemia therapeutic approaches

**DOI:** 10.18632/oncotarget.2947

**Published:** 2014-12-10

**Authors:** Ângela Fernandes, Maria M. Azevedo, Olga Pereira, Belém Sampaio-Marques, Artur Paiva, Margarida Correia-Neves, Isabel Castro, Paula Ludovico

**Affiliations:** ^1^ Life and Health Sciences Research Institute (ICVS), School of Health Sciences, University of Minho, Braga, Portugal; ^2^ ICVS/3B's - PT Government Associate Laboratory, Braga/Guimarães, Portugal; ^3^ Blood and Transplantation Center of Coimbra, Portuguese Institute of Blood and Transplantation, Coimbra, Portugal

**Keywords:** acute myeloid leukemia (AML), macroautophagy, ubiquitin-proteasome system (UPS), AMPK pathway, chemotherapeutic agents

## Abstract

The therapeutic strategies against acute myeloid leukemia (AML) have hardly been modified over four decades. Although resulting in a favorable outcome in young patients, older individuals, the most affected population, do not respond adequately to therapy. Intriguingly, the mechanisms responsible for AML cells chemoresistance/susceptibility are still elusive. Mounting evidence has shed light on the relevance of proteolytic systems (autophagy and ubiquitin-proteasome system, UPS), as well as the AMPK pathway, in AML biology and treatment, but their exact role is still controversial. Herein, two AML cell lines (HL-60 and KG-1) were exposed to conventional chemotherapeutic agents (cytarabine and/or doxorubicin) to assess the relevance of autophagy and UPS on AML cells’ response to antileukemia drugs. Our results clearly showed that the antileukemia agents target both proteolytic systems and the AMPK pathway. Doxorubicin enhanced UPS activity while drugs’ combination blocked autophagy specifically on HL-60 cells. In contrast, KG-1 cells responded in a more subtle manner to the drugs tested consistent with the higher UPS activity of these cells. In addition, the data demonstrates that autophagy may play a protective role depending on AML subtype. Specific modulators of autophagy and UPS are, therefore, promising targets for combining with standard therapeutic interventions in some AML subtypes.

## INTRODUCTION

Acute myeloid leukemia (AML) is a highly heterogeneous clonal disorder comprising the most common acute leukemias in adults [1-3]. This disease is characterized by an impairment in myeloid cellular differentiation with accumulation of immature myeloid progenitor cells in the bone marrow [1-3]. Hence, AML heterogeneity is reflected by the diversity of myeloid precursors susceptible to malignant transformation.

To date, AML therapeutic schemes result in favorable outcomes for young patients but, among old individuals have limited application, commonly due to the high toxicity [4, 5]. Of note, despite the efforts to understand AML and the considerable progress in the therapy, this disorder is still fatal to two-thirds of young adults and 90 % of older adults that develop the disease [6]. Thus, the elucidation of the mechanism(s) underlying leukemia cell's response to chemotherapy is of utmost importance for the development of new strategies, targeting specific molecular alterations that perturb AML cell's survival pathways.

The conventional therapy for AML patients during induction relies mainly on high doses of cytarabine in combination with an anthracycline, such as doxorubicin [7]. Both drugs induce a DNA damage response [8, 9] that once activated may lead to LKB1 (Liver kinase B1) and AMPK (AMP-activated protein kinase) activation [10-12]. The LKB1-AMPK pathway is a key sensor of the cellular energy status that is also responsible for the activation of critical survival mechanisms. This pathway has been implicated in cancer cell biology [13] mainly as a tumor suppressor axis [14-19]. In hematopoietic cells, LKB1-AMPK has been demonstrated to be critical for the maintenance of energy homeostasis [20]. Therefore, its modulation is viewed as a promising novel treatment option for some types of hematological malignancies [13, 14, 20, 21]. Interestingly, LKB1-AMPK is also involved in the regulation of protein degradation through direct and indirect mechanism affecting the two main proteolytic systems, ubiquitin-proteasome system (UPS) and macroautophagy (hereafter called autophagy) [22, 23]. The relevance of both proteolytic systems on cancer biology and particularly on hematological malignancies has been widely recognized [24-27]. Inhibition of UPS is already an attractive target for therapeutic interventions in different cancers including hematological malignancies. Bortezomib, a reversible inhibitor of the chymotrypsin-like activity of UPS, was already approved for the treatment of relapsed and refractory multiple myeloma and mantle cell lymphoma [28-30]. Although it has been shown that AML cells are more susceptible to UPS inhibition than non-neoplastic cells, their response is dependent on the AML subtype [31].

Importantly, autophagy may act as a “double-edged sword” in cancer [32]. In early tumorigenesis, it seems to be cancer preventing, however, in an established tumor, autophagy might help cancer cells to survive under stress. Under prolonged cytotoxic treatments, autophagy enhancement may act as a cancer resistance mechanism [33] and can be activated in response to DNA damaging agents, counteracting their action by avoiding cell death [26]. Particularly in AML chemotherapy, autophagy was described as reducing the activity of daunorubicin, obatoclax and vitamin D3 [34-36]. Furthermore, recent evidence suggests that autophagy acts as a pro-survival signal in t(8;21) AML cells [37] and, in accordance, a dual inhibition of mTORC1 and mTORC2 leads to an autophagy induction with consequent increase of AML cells survival [38]. In contrast, AZD8055, a dual inhibitor of mTORC1 and mTORC2 and a strong inducer of autophagy, decreases AML blast cell proliferation suggesting that autophagy might be contributing for cell death [39]. In agreement, the reduction of TRAF6 protein, promoted by bortezomib or protein knockdown, is associated to autophagy induction and subsequently with apoptosis of AML cells [40]. Therefore, although the autophagic process is emerging as a crucial player and a promising therapeutic target in AML [26], its exact role is still controversial.

Autophagy has also been shown to be regulated by the LKB1-AMPK pathway. This energy sensing pathway is known to be involved in complex interplays with mTORC1 and both have been suggested to have opposite effects on the regulation of this catabolic process [41].

Clinical trials using autophagy and UPS inhibitors for the treatment of different hematological malignancies are already ongoing, however, the results are still inconclusive and further investigation is required (reviewed in [42]).

Given the still controversial role of UPS, autophagy and AMPK pathway on survival/death of AML blasts, this work focused on evaluating the relevance of these processes on AML cells’ response to anti-leukemia agents. The results obtained with two AML cell lines highlighted the heterogeneous and specific nature of the different AML subtypes, in what concerns the relevance of AMPK and the proteolytic systems in response to chemotherapeutic agents. Chemotherapy regime promotes in HL-60 cells an increase of UPS activity that leads to extensive AMPK degradation, which among others effects, results in autophagy inhibition. In KG-1 cells these effects are discreet and most probably related to the specific features displayed by these cells such as high UPS activity. Altogether, the results show that these survival pathways are promising targets for therapeutic intervention in some AML subtypes and have to be considered in association with standard chemotherapy.

## RESULTS

### Toxic effects of cytarabine and doxorubicin on AML cell lines

**Figure 1:**
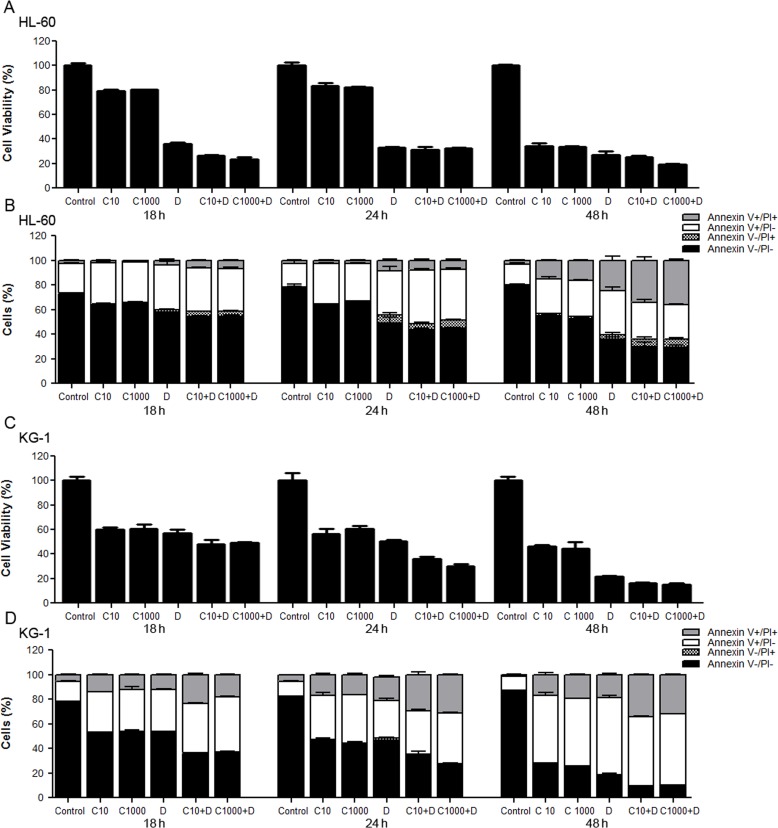
Toxicity and antitumor effects of cytarabine and doxorubicin on AML cell lines HL-60 and KG-1 cells were incubated for 18 h, 24 h and 48 h with cytarabine and/or doxorubicin. Cellular viability was assessed using the MTS and annexin V/PI assays. The results were determined using the non-treated cells as control (100 % of viability) and presented as mean+/−SEM of, at least, six biological replicates. One-way ANOVA and Turkey's Multiple Comparison Test were used to compare the non-treated group with the treated groups and within treated groups in the MTS assay. Annexin V/PI data was analyzed by two-way ANOVA and Bonferroni post hoc test. Significant differences were obtained between cells untreated vs cells treated and between cells individually treated with cytarabine or doxorubicin vs cells treated with the combination of both antileukemia agents. Treatment of cells with different concentrations of cytarabine did not present statistically significant differences in cell viability. (A), (C) - HL-60 and KG-1 cells viability determined by MTS assay, respectively; (B), (D) - HL-60 and KG-1 cells viability determined by annexin V/PI assay, respectively. Legend: C - cytarabine, D - doxorubicin, C+D - cytarabine combined with doxorubicin. Cytarabine: 10 μM or 1000 μM to HL-60 and KG-1 cells; Doxorubicin: 3 μM to HL-60 cells and 2 μM to KG-1 cells.

To get new insights into the mechanisms underlying resistance/susceptibility of AML cells to cytarabine and doxorubicin, human AML cells derived from patients diagnosed with FAB M2 (HL-60 cell line) and FAB M6 - erythroleukemia (KG-1 cell line) were exposed to these chemotherapeutic agents for 18 h, 24 h and 48 h. Two concentrations of cytarabine were used: 10 μM, the most commonly used cytarabine concentration for human AML cell lines in *in vitro* assays [43-45] and 1000 μM, to mimic chemotherapeutic regimens consisting of high cytarabine concentrations [46, 47]. Regarding doxorubicin, the half maximal inhibitory concentrations (IC_50_) were used (Table 1). The results showed that cytarabine alone only has a drastic impact on AML cells survival for longer incubation periods (Figure 1), which is in agreement with the commonly used 7 days perfusion therapeutic schemes. Moreover, for the treatment time periods analyzed, the 100-fold increase in the cytarabine concentration had no effect on HL-60 or KG-1 cells’ death rate, measured by MTS and annexin V/PI assays (Figure 1). Concerning doxorubicin, the concentrations chosen induced around 40 to 60 % cell death in both cell lines (Figure 1). As expectable, exposure of HL-60 and KG-1 cells to the combination of the two chemotherapeutic agents for the same incubation periods resulted in enhanced loss of cell viability in a time-dependent manner, compared to the individual treatments (Figure 1).

Of note, the comparison of the cell survival percentages obtained by MTS and annexin V/PI assays showed a good correlation between both methodologies for KG-1 cells (Figure 1C and Figure 1D) but not for HL-60 cells, particularly in treatment conditions involving doxorubicin (Figure 1A and Figure 1B). Previous studies reported that doxorubicin affects mitochondrial activity on HL-60 cells [48], which may be responsible for the different results obtained with the two methods on this cell line and highlight the need to carefully interpret the data using MTS to evaluate cell viability in this particular condition and the usefulness of using more than one assay to evaluate cell viability/survival.

**Table 1:** Concentrations of the drugs - cytarabine (C), doxorubicin (D), bortezomib (B), bafilomycin A1 (B A1) and compound C (CC) - used in HL-60 and KG-1 cell lines

Concentrations (μM)		
	HL-60	KG-1
Cytarabine (C)	10 or 1000	10 or 1000
Doxorubicin (D)	3	2
Bortezomib (B)	0.02	0.01
Bafilomycin A1 (B A1)	0.01	0.01
Compound C (CC)	2.5	0.5

### Combination of antileukemia agents induces DNA damage and leads to AMPK degradation on AML cell lines

**Figure 2:**
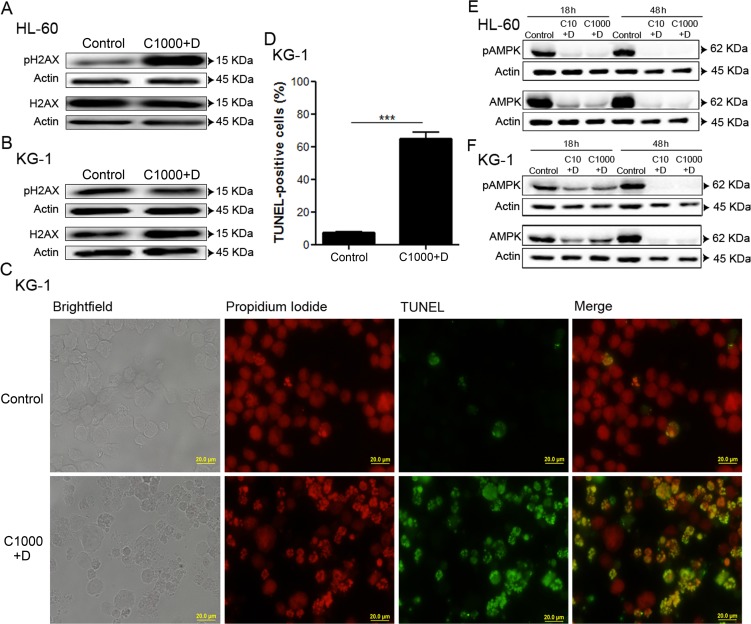
Combination of antileukemia agents induces DNA damage and leads to AMPK degradation on AML cell lines HL-60 and KG-1 cells were incubated, for 18 h, with cytarabine and doxorubicin. After the treatment period, DNA damage was evaluated by immunoblotting analysis of phosphorylated (Ser139) and total H2AX protein levels (A, B). Terminal dUTP Nick-End Labeling (TUNEL) staining was performed (green fluorescence) for KG-1 cells and the samples were counter-stained with the DNA dye Propidium Iodide (red fluorescence). A minimum of 300 cells were counted per condition and the percentage of TUNEL positive cells was determined. The results were obtained by comparing the treated with non-treated cells and presented as mean+/−SEM of three intra-assay replicates. Student's *t*-test was used to compare the non-treated group with the treated group. ***p <0.001. (C) – Representative images of TUNEL assay for KG-1 cells. (D) – Quantification of the TUNEL positive cells. AMPK activation was evaluated by immunoblotting analysis of phosphorylated (Thr172) and total AMPK protein levels after 18 h or 48 h of treatment (E, F). Legend: C+D - cytarabine combined with doxorubicin. Cytarabine: 10 μM or 1000 μM to HL-60 and KG-1 cells; Doxorubicin: 3 μM to HL-60 cells and 2 μM to KG-1 cells.

To evaluate the impact of antileukemia agents (cytarabine and doxorubicin) on DNA damage, we assessed the levels of phosphorylated (Ser139) and total histone H2AX protein by immunoblotting analysis, an important marker of DNA damage response activation [49]. The data showed that, in HL-60 cells, the combination of the antileukemia agents induced a marked increase of H2AX phosphorylation, when compared with untreated cells (Figure 2A). In contrast, no major alterations of H2AX phosphorylation were observed when KG-1 cells were exposed to the same treatment (Figure 2B). In fact, KG-1 cells displayed high basal levels of H2AX phosphorylation (Figure 2B), a phenomenon also documented by Boehrer *et al*. upon exposure of KG-1 cells to different doses of irradiation [50]. Therefore, to further elucidate whether the combination of cytarabine and doxorubicin induced DNA damage in KG-1 cells, a Terminal dUTP Nick-End Labeling (TUNEL) assay was performed. The results clearly showed an increase in the percentage of TUNEL positive cells (from about 8 % in untreated cells to 65 % in cells treated with chemotherapy agents), confirming the induction of DNA damage by cytarabine and doxorubicin in KG-1 cells (Figure 2C and Figure 2D).

To investigate whether DNA damage response lead to AMPK activation [10-12], the levels of phosphorylated AMPK (Thr172, the residue phosphorylated in response to DNA damage) and total AMPK protein were determined by immunoblotting analysis. Our data demonstrated that HL-60 cells presented high basal levels of total and phosphorylated AMPK, in comparison to KG-1 cells (Figure 2E and Figure 2F), which suggests a constitutive activation of this pathway in HL-60 cells.

Interestingly, the cytarabine plus doxorubicin treatment resulted in a marked decrease of total AMPK protein levels in both cell lines, when compared with untreated cells. Of note, such decrease was more evident in HL-60 cells (Figure 2E and Figure 2F). According to the literature, the AMPK pathway is critically involved in the regulation of proliferation and survival of leukemia cells [14]. In agreement, our data also suggests AMPK as an important survival pathway in AML cells that seems to be targeted by the combination of cytarabine and doxorubicin.

### Doxorubicin induces UPS activation on AML cell lines

**Figure 3:**
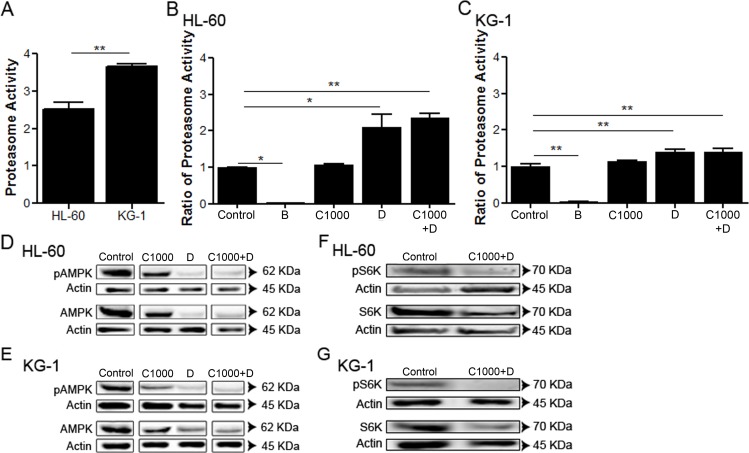
Doxorubicin induces UPS activation on AML cell lines HL-60 and KG-1 (A) UPS basal levels were quantified by the measurement of the chymotrypsin-like activity. Additionally, HL-60 (B) and KG-1 (C) cells were incubated, for 18 h, with bortezomib (an UPS inhibitor used as negative control), cytarabine and/or doxorubicin. After the treatment period, the UPS activity was quantified using the approach referred above. The results were obtained by comparing the treated with non-treated cells and mean+/−SEM of three intra-assay replicates are shown. One-way ANOVA and Turkey's Multiple Comparison Test were used to compare the non-treated group with the treated groups. *p <0.05; **p <0.01. Additionally, AMPK and S6K (mTORC1 substrate) activation were evaluated by immunoblotting analysis of phosphorylated (Thr172) and total AMPK protein levels and phosphorylated (Thr389) and total S6K protein levels, respectively, after 18 h of treatment on the HL-60 (D, F) and KG-1 (E, G) cell lines. The arrangement of the AMPK gels is represented separately due to the presence of other conditions between the bands of interest. Nevertheless, all conditions were assessed in the same gel. Legend: B – bortezomib, C – cytarabine, D – doxorubicin, C+D -cytarabine combined with doxorubicin. Bortezomib: 0.02 μM to HL-60 cells and 0.01 μM to KG-1 cells; Cytarabine: 1000 μM to HL-60 and KG-1 cells; Doxorubicin: 3 μM to HL-60 cells and 2 μM to KG-1 cells.

Recent studies have highlighted that UPS might directly or indirectly regulate AMPK activity [51]. To elucidate whether UPS was responsible for the observed AMPK degradation, under chemotherapeutic agents’ exposure (Figure 2E and Figure 2F), UPS activity was determined. The results indicated that KG-1 cells present a higher basal level of UPS activity, when compared to HL-60 cells (Figure 3A). However, in the presence of doxorubicin (alone or combined with cytarabine), the condition where the AMPK levels are evidently reduced (Figure 2E, Figure 2F, Figure 3D and Figure 3E), a significant increase in UPS activity was detected in both cell lines (Figure 3B and Figure 3C). Of note, the increased UPS activity was much more pronounced in HL-60 cells than in KG-1 cells, the cell line that displayed the higher basal levels of UPS activity (Figure 3A, Figure 3B and Figure 3C). Furthermore, the observation of less AMPK degradation in KG-1 cells treated with the chemotherapeutic agent, when compared with HL-60 cells in the same conditions, could be associated with the lower enhancement of UPS activity observed when KG-1 cells are subjected to doxorubicin (Figure 3B and Figure 3C). Consistently with the specific degradation of AMPK by UPS, our results showed that the combination of the antileukemia agents also promoted a decrease of the protein levels of the mTOR substrate S6K in both HL-60 and KG-1 cell lines (Figure 3F and Figure 3G). Altogether, the results obtained showed that AML cell lines have distinct basal levels of UPS activity and that doxorubicin is effectively targeting AMPK for degradation.

### Combined antileukemia agents impact on autophagy activity of AML cell lines, particularly HL-60 cell line

**Figure 4:**
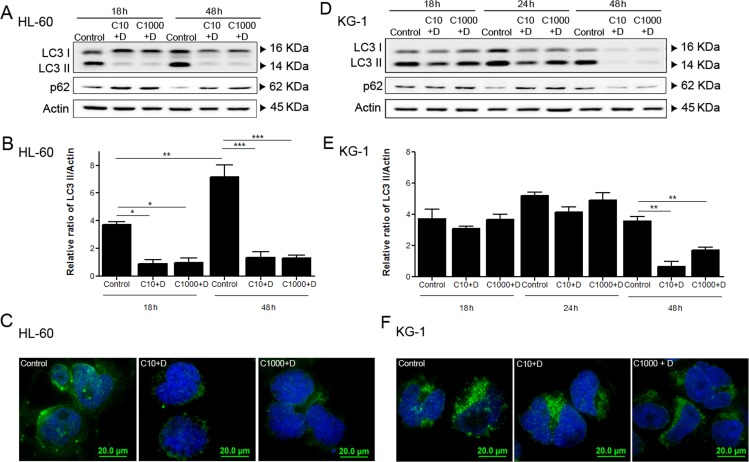
Combined antileukemia agents impact on autophagy activity of AML cell lines, particularly in HL-60 cells HL-60(A, B) and KG-1 (D, E) cells were incubated for 18 h, 24 h (only for KG-1 cells) and 48 h, with cytarabine and doxorubicin and after the treatment period, the autophagy activity was assessed using immunoblotting analysis of LC3 processing (I and II) and p62. All samples were incubated 2 h before the end of the treatment with bafilomycin A1 (10 nM), in order to block the autophagy flow and to allow the accumulation of LC3-II. Densitometric analysis of LC3 protein levels in HL-60 (B) and KG-1 (E) cells was performed using the ratio LC3II/Actin. The bands were quantified in Quantity One® software and the results represent six independent biological replicates. One-way ANOVA and Turkey's Multiple Comparison Test were used to compare the all the group conditions.*p<0.05; **p<0.01; ***p<0.001. Additionally, LC3 A/B-I/II puncta levels were assessed by immunofluorescence assay after 18 h of treatment. HL-60 (C) and KG-1 (F) cells were staining to Goat LC3 anti-Rabbit IgG antibody (green fluorescence) and samples were counter-stained with the DNA dye DAPI (blue fluorescence). Representative images of immunofluorescence assay are presented. Bar=20 μm. Legend: C+D - cytarabine combined with doxorubicin. Cytarabine: 10 μM and 1000 μM to HL-60 and KG-1 cells; Doxorubicin: 3 μM to HL-60 cellsand 2 μM to KG-1 cells.

Given the increased UPS activity observed in response to doxorubicin in AML cells (Figure 3B and Figure 3C), we investigated the involvement of autophagy, the other main proteolytic system, on AML cell's response to chemotherapy. For such purpose, autophagy flux was assessed by the immunoblotting analysis of LC3 processing and of p62 levels (also known as SQSTM1), an autophagy substrate [52]. The results showed that both cell lines present similar basal autophagic activity levels that increase over treatment time (controls from Figure 4A, Figure 4B, Figure 4D and Figure 4E). Interestingly, a similar increase in the AMPK basal levels over the course of the experiment was also observed (Figure 2E and Figure 2F), which is consistent with the central role of the AMPK pathway on the regulation of autophagy [53]. As expected, a decrease in p62 levels was also observed in a time dependent manner (Figure 4A and Figure 4D). The cytarabine/doxorubicin combined treatment caused a drastic decrease of autophagic levels in HL-60 cells, observed by the clear reduction of LC3 II levels in the immunoblotting analysis (Figure 4A and Figure 4B). This observation is supported by the evident increase of p62 levels and decrease of LC3 puncta observed in these cells (Figure 4A and Figure 4C). In contrast, for KG-1 cells, the combination of the antileukemia agents did not promote any major impact on autophagy up to 24 h of treatment (Figure 4D and Figure 4F). Nevertheless, a clear reduction of both LC3 I and LC3 II levels, as well as of p62 levels, was evident after 48 h of treatment with cytarabine and doxorubicin (Figure 4D and Figure 4E), a phenomenon that was not observed in HL-60 cells (Figure 4A and Figure 4B).

The data showed that the combination of cytarabine and doxorubicin is efficient in targeting and inhibiting autophagy in HL-60 cells but they can only promote a similar effect on KG-1 cells for long time period (48 h, Figure 4D and Figure 4E), highlighting that the behavior of the proteolytic systems in response to chemotherapy is highly dependent on AML subtype.

### Modulation of autophagy affects AML cells viability

**Figure 5:**
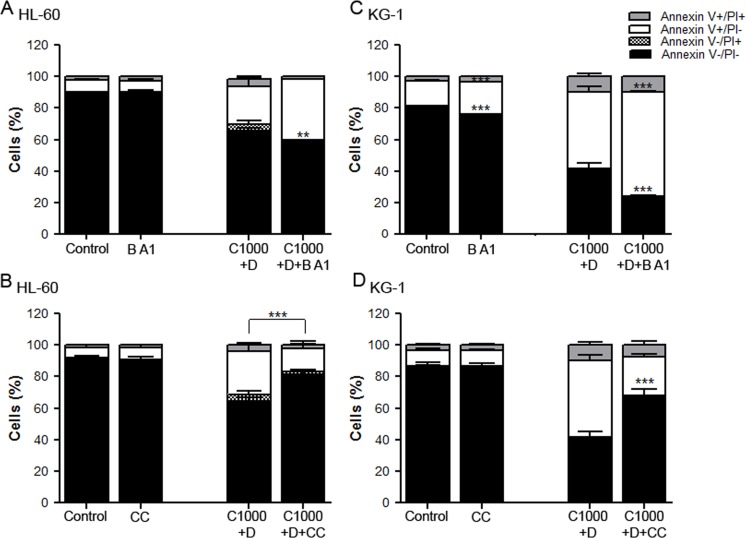
Modulation of autophagy affects AML cells viability HL-60 (A, B) and KG-1 (C, D) cells were incubated, for 18 h, with cytarabine and doxorubicin, combined with bafilomycin A1, an autophagy inhibitor and with compound C, an indirect autophagy activator (pre-incubation with compound C during 24 h). After the treatment, cells viability was assessed using annexin V/PI assays. The results were obtained using the non-treated cells as control (100 %) and mean+/−SEM of, at least, six biological replicates are shown. Annexin V/PI data was analyzed by two-way ANOVA and Bonferroni post hoc test. ***p <0.001. Legend: B A1 – bafilomycin A1; CC – compound C; C+D - cytarabine combined with doxorubicin. Bafilomycin A1: 10 nM to HL-60 and KG-1 cells; Compound C: 2.5 μM to HL-60 cells and 0.5 μM to KG-1 cells; Cytarabine: 1000 μM to HL-60 and KG-1 cells; Doxorubicin: 3 μM to HL-60 cells and 2 μM to KG-1 cells.

To further clarify the role of autophagy on AML cells’ response to chemotherapy, the impact of its modulation on the viability of HL-60 and KG-1 cells exposed to cytarabine and doxorubicin was determined. For such, bafilomycin A1, an autophagy inhibitor, and compound C, an efficient pharmacological AMPK inhibitor [54] that is also an indirect autophagic activator were used. The results showed that addition of bafilomycin A1 together with cytarabine and doxorubicin has a discrete effect on the viability of AML cells, although it is more pronounced on KG-1 cells (Figure 5A and Figure 5C). Nevertheless, the combination of compound C, an autophagy inducer/activator, with cytarabine and doxorubicin promoted a statistically significant increase in HL-60 and KG-1 cells’ viability (Figure 5B and Figure 5D). Although the use of indirect autophagy modulators has important limitations, altogether the results clearly showed that induction of autophagy promotes cell survival.

## DISCUSSION

Cytarabine and doxorubicin are two chemotherapeutic agents currently used in the treatment of AML [7, 55]. This therapeutic scheme has hardly progressed over the last 40 years in spite of its low efficiency rates and limited application among old individuals and high levels of resistance displayed by AML cells to the drugs [5]. Cytarabine and doxorubicin are well known inducers of DNA damage [56, 57] and the impact of these agents on DNA damage was confirmed on HL-60 and KG-1 cells in the conditions described. Exposure of both cell lines to the combination of the two chemotherapeutic agents induced a clear enhancement of H2AX phosphorylation (in HL-60 cells) and a drastic increase in the percentage of TUNEL positive cells (in KG-1 cells). KG-1 cells were shown to display a high constitutive level of H2AX phosphorylation. The role of histone H2AX in malignant transformation/cancer development is still not totally clear and novel functions for phosphorylation of histone H2AX, in addition to DNA damage repair, have already been shown, including the induction of its phosphorylation through proliferation signaling pathways [58], which could occur in the case of KG-1 cells.

Although most anticancer therapies rely mainly on agents that affect the machinery required for DNA replication to induce cell death [59], over the last years, different studies have highlighted that chemotherapeutics-induced DNA damage can also lead to the activation of AMPK, a master regulator of cellular energy homeostasis [15-17]. Comparison of basal AMPK activity levels of the two cell lines studied suggested an association between AMPK and the tumorigenicity of the HL-60 cells. Notably, the cytarabine and doxorubicin treatment induced a marked reduction of total AMPK levels that is consistent with the increased activity of proteasome. This phenomenon is clearly evident in HL-60 cells but not in KG-1 cells. Therefore, our data clearly rules out the hypothesis of AMPK activation being an AML cell survival mechanism activated by the chemotherapeutic agents but instead suggests that it could be part of the tumorigenic process of HL-60 cells.

Previous studies reported that UPS could regulate AMPK activity by inducing its ubiquitination and consequent degradation [51]. Our data is consistent with this hypothesis since an increase in UPS activity was associated with the AMPK degradation observed on both cell lines when exposed to doxorubicin. Of note, the lower AMPK degradation in KG-1 cells treated with the chemotherapeutic agent, when compared with HL-60 cells in the same conditions, could then be associated with the lower enhancement of UPS activity observed when KG-1 cells are subjected to doxorubicin. Moreover, the basal UPS activity of KG-1 cells was found to be higher than in HL-60 cells in accordance with the higher content of KG-1 cells in 20S proteasome previously described [31]. Altogether, the data suggests that doxorubicin leads to increased UPS activity, resulting in the degradation of critical cellular signal transducers and effectors such as AMPK and S6K, a downstream target of mTORC1 that in turn could also be regulated by the AMPK pathway [60]. These findings are highly relevant in the context of the antitumoral action of doxorubicin, suggesting that AMPK and mTORC1 are important targets of this drug. The results also suggest that UPS activity is important on AML cell's response to doxorubicin and that cells with higher UPS activity, KG-1 cells, are more resistant to doxorubicin (as shown by the higher IC_50_ value).

Autophagy is another crucial cellular proteolytic system of eukaryotic cells that, together with UPS, plays a crucial role on protein catabolism and cellular homeostasis [61]. Our findings, when assessing the effect of the two chemotherapeutics’ combination in the autophagic activity, clearly demonstrated the differential response of the two cell lines analyzed. The exposure of HL-60 cells to cytarabine and doxorubicin caused an autophagy blockage, accompanied by a drastic reduction of the AMPK levels. In the KG-1 cell line, however, the combination of these drugs did not significantly impact the autophagic activity, neither led to AMPK degradation in such a significant manner.

The exact role of autophagy in cancer development/treatment is still controversial. In AML disorders, recent evidence suggests that autophagy acts as a pro-survival signal in t(8;21) AML cells [37], however the exact role of this process in the different subtypes of this hematologic malignancy is still unclear. Our data on autophagy after treatment and the modulation of this proteolytic process clearly demonstrates the crucial role of autophagy as a pro-survival mechanism in AML cells. Nevertheless, autophagy seems to be targeted by cytarabine and doxorubicin combination in HL-60 cells only. Our results imply that, in this cell line, the combination of drugs leads to autophagy blockage or autophagy players’ and regulators’ degradation. In addition, the data clearly highlights the relevance that proteolytic systems might have on cells response to chemotherapy and the heterogeneity of the response of AML subtypes to conventional chemotherapy. In accordance, examples of heterogeneity in responses to drugs, leading to the activation of different cell death mechanisms, including autophagy, have been recently described as being dependent on the leukemia subtype, in T-cell acute lymphoblastic leukemia [62].

In summary, this study highlights the relevance of the proteolytic systems and one of their major regulators, AMPK, on AML cells response to chemotherapy, which suggests that specific modulators of AMPK activity and the autophagy and UPS systems may be promising targets for therapeutic intervention in AML. The association of such modulators to standard therapeutic strategies could actually allow the reduction of the chemotherapeutics’ doses, therefore reducing their toxicity. Identification of specific molecular mutations or altered mechanisms and appropriate use of this knowledge to the development of targeted therapies is recent in the treatment strategy of AML. The best studied potential targets are FMS like tyrosine kinase 3 (FLT3), RAS/RAF/MEK/ERK and Janus kinase (JAK-2) [63]. Based on the findings described in this paper, the heterogeneous response of the AML subtypes to the therapy commonly used corroborates with the strong rationale for the development of different and specific therapeutic schemes to apply to AML patients, dependent on the molecular abnormalities of each subtype. Specifically, our data suggests the AMPK pathway and both proteolytic systems might be strong potential candidates. Nevertheless, future studies to dissect and confirm this data should be performed in primary AML samples.

## METHODS

### Cell culture

The HL-60 (FAB M2) and KG-1 (erythroleukemia – FAB M6) cell lines were obtained from the German Collection of Microorganisms and Cell cultures (DSMZ^®^ - Deutsche Sammlung von Mikroorganismen und Zellkulturen - German). Cells were cultured in RPMI 1640 medium (Biochrom^®^ - Merck Millipore) supplemented with 10 % heat-inactivated fetal bovine serum – FBS - (Biochrom^®^ - Merck Millipore) and 1 % antibiotic-antimycotic mixture (Invitrogen^®^) in a humidified, 37°C, 5 % CO_2_ atmosphere. Cells in exponential phase of growth (passages 5 to 20) were used.

### Treatments

Cytarabine, doxorubicin and compound C were purchased from Sigma-Aldrich^®^ and were dissolved in dH_2_O. Bafilomycin A1 was also purchased from Sigma-Aldrich^®^ but prepared in DMSO. Bortezomib was obtained from Santa Cruz^®^ and was prepared in dH_2_O. The concentrations used for each drug are described in Table 1.

### Measurement of cell survival – MTS assay

Cell viability of HL-60 and KG-1 cells was assessed using the Cell Titer 96 Aqueous One Solution Cell Proliferation Assay (MTS), acquired from Promega^®^. HL-60 and KG-1 cell lines were plated at 350.000 cells/700 μl per well and exposed to the different drug combinations. The number of viable cells in culture was determined at 18 h, 24 h and 48 h of treatment exposure (or control). 100 μl of the cells’ suspension was then transferred to 96-well plates and 10 μL of MTS solution (1.90 mg/ml) were added to each well followed by incubation in a humidified, 37°C, 5 % CO_2_ atmosphere. Absorbance at 490 nm was recorded after 2 h of incubation. Blank controls detecting cell-free RPMI + drug and RPMI absorbances were performed in parallel. At least six biological replicates were prepared.

### Measurement of cell viability – Annexin V and PI analysis

HL-60 and KG-1 cell lines were plated at 350.000 cells/700 μl per well and collected at 18 h, 24 h and 48 h of treatment (or control). The cells were then washed with 800 μl of PBS and 100 μL of binding buffer was added to each sample. An incubation with 5 μL of annexin V (BD Biosciences^®^) and with 10 μL of Propidium iodide (PI) at 50 μg/ml (Invitrogen^®^) was performed for 15 min at room temperature, in the dark, and 200 μL of binding buffer were added once again. PI signals were measured using the FACS LSRII flow cytometer (BD Biosciences^®^) with a 488 nm excitation laser. The annexin V signal was collected through a 488 nm blocking filter, a 550 nm long-pass dichroic with a 525 nm band pass. Signals from 10.000 cells/sample were captured and FACS Diva was used as the acquisition software. Results analysis was performed using the FlowJo 7.6 (Tree Star^®^) software. The results represent, at least, six biological replicates.

### Western blot analysis

Protein extraction was performed with 50 μL of lysis buffer (1 % NP-40, 500 mM Tris HCL, 2.5 M NaCl, 20 mM EDTA, Phosphatase and Protease inhibitors - from Roche^®^ -, at pH 7.2) 18 h, 24 h and 48 h after HL-60 and KG-1 cell lines were exposed to treatment (or control). 20 μg of the total protein were resolved in a 12 % SDS gel and transferred to a Nitrocellulose membrane for 7 or 15 min in Trans-Blot Turbo^®^ transfer system. Membranes were blocked in phosphate-buffered saline (PBS) with 0.1 % tween 20 (PBS-T) containing 5 % skim milk Molico^®^ and afterwards incubated overnight at 4°C, with the polyclonal primary antibodies at 1:1000 in 1 % BSA (Bovine Serum Albumin) - Rabbit anti-histone H2AX Antibody; Rabbit anti-phospho-histone H2AX (Ser139) Antibody; Rabbit anti-AMPKα Antibody; Rabbit anti-phospho-AMPKα (Thr172) Antibody; Rabbit anti-p70 S6K Antibody; Rabbit anti-phospho-p70 S6K Antibody (Thr389); Rabbit anti-LC3A/B Antibody (all from Cell-Signaling^®^); Rabbit anti-p62 Antibody (Sigma-Aldrich^®^) and Mouse anti-Actin antibody (Millipore^®^). After washing with tris-buffered saline-tween (TBS-T), membranes were incubated with the corresponding secondary antibodies - IgG anti-Mouse for Actin (Chemicon International^®^) and IgG anti-Rabbit for all the others (Cell-Signaling^®^), at 1:5000 in 1 % skim milk for 2 h at room temperature. Protein levels were detected after incubation with SuperSignal^®^ West Femto Maximum Sensitivity Substrate (Thermo Scientific^®^) or Clarity^®^ Western ECL Substrate (Bio-Rad^®^). Digital images of the western blotting were obtained in a ‘ChemiDoc XRS System’ (Bio-Rad^®^) with Quantity One (Bio-Rad^®^) software. At least, six independent biological replicates were performed.

### TUNEL analysis

The cytospin technique was performed to fixe 50.000 HL-60 or KG-1 cells/plate, after 18 h of treatment (or control). Cells were fixed (PFA 4 %), washed and permeabilized with 0.1 % Triton X-100 in 0.1 % sodium citrate for 7 min on ice. The Terminal dUTP Nick-End Labeling (TUNEL) analysis was performed using the “In Situ Cell Death Detection” kit, Fluorescein (Roche®) following the manufacter‘s instructions. Cells were also stained with PI (Invitrogen^®^), binding DNA regions. Three independent biological replicates were used. An epifluorescence microscope (BX61 microscope with an Olympus DP70 camera) was used for slide visualization and then the images were analyzed with ImageJ^®^ Software (National Institutes of Health).

### Proteolytic activity of Ubiquitin-proteasome system (UPS)

“Proteasom-Glo^TM^ Cell-Based Assays” (Promega^®^) was used to measure the proteolytic activity of the UPS. It comprises a luminescent assay that measures the chymotrypsin-like activity associated with the proteasome complex in cells [64]. Chymotrypsin-like (SUC-LLVY-Glo subs) was chosen as a proteasome subtract. Proteasome-Glo™ Cell-Based Reagents were both prepared and equilibrated at 22°C for 30 min before use. The luminescence was measured in a luminometer “Fluoroskan Ascent FL” by ThermoScientific. 350.000 cells (HL-60 or KG-1) were diluted in 700 μl of RPMI 1640 medium containing 10 % FBS and 1 % antibiotic with treatments and equilibrated in an incubator with controlled % of CO_2_ (5 %), temperature (37°C) and humidity. After 18 h of treatment (or control), 25.000 cells were plated in a 96-well opaque plate, comprising a volume of 100 μl. This was combined with “Proteasom-Glo^TM^Cell-Based Assays” (100 μl), the assay plate was equilibrated at 22°C, and after incubation during 5 to 30 min, a luminescent signal was obtained.

### Immunofluorescence staining

HL-60 or KG-1 cells (30.000 cells/plate) were resuspended in PBS, after 18 h under treatment, and were fixed in a slide by cytospin technic. After this, fixation was performed in PFA 2 %. Cells were then washed, permeabilized and blocked with 4 % BSA in PBS 0.05 % Tween. Incubation with primary antibody, Rabbit anti-mouse LC3 A/B (Cell-Signaling^®^), was executed overnight, at 4°C. Goat anti-Rabbit IgG Alexa Fluor 488 - green-fluorescent dye - (Molecular Probes^®^) was used as secondary antibody. Cells were also stained with DAPI (4′,6-diamidino-2-phenylindole) that binds regions in DNA, marking the cell nuclei. Three biological replicates were used. An epifluorescence microscope (BX61 microscope with an Olympus DP70 camera) was used to slide visualization and then images were analyzed with ImageJ^®^ Software (National Institutes of Health).

### Statistical analysis

All data is reported as the mean ± standard error of the mean (SEM). Statistical analysis was performed using the student's *t*-test and the one-way ANOVA test. Multiple comparison test was used as Post-Hoc test to denote significant differences between groups untreated, groups subjected to drugs and respective combinations. Additionally, the two-away ANOVA and Bonferroni post hoc tests were also used to compare the non-treated group with the treated groups and respective combinations for annexin V/PI approaches. A p-value lower than 0.05 was assumed to denote a significant difference.
